# Development of Moderate Intensity Focused Ultrasound (MIFU) for Ocular Drug Delivery

**DOI:** 10.34133/2022/9840678

**Published:** 2022-06-08

**Authors:** Alejandra Gonzalez-Calle, Runze Li, Isaac Asante, Juan Carlos Martinez-Camarillo, Stan Louie, Qifa Zhou, Mark S. Humayun

**Affiliations:** ^1^USC Ginsburg Institute for Biomedical Therapeutics, University of Southern California, Los Angeles California, USA; ^2^USC Roski Eye Institute, Keck School of Medicine, University of Southern California, Los Angeles California, USA; ^3^Department of Biomedical Engineering, University of Southern California, Los Angeles California, USA; ^4^USC School of Pharmacy, University of Southern California, Los Angeles California, USA

## Abstract

The purpose of this study is to develop a method for delivering antiinflammatory agents of high molecular weight (e.g., Avastin) into the posterior segment that does not require injections into the eye (i.e., intravitreal injections; IVT). Diseases affecting the posterior segment of the eye are currently treated with monthly to bimonthly intravitreal injections, which can predispose patients to severe albeit rare complications like endophthalmitis, retinal detachment, traumatic cataract, and/or increased intraocular. In this study, we show that one time moderate intensity focused ultrasound (MIFU) treatment can facilitate the penetration of large molecules across the scleral barrier, showing promising evidence that this is a viable method to deliver high molecular weight medications not invasively. To validate the efficacy of the drug delivery system, IVT injections of vascular endothelial growth factor (VEGF) were used to create an animal model of retinopathy. The creation of this model allowed us to test anti-VEGF medications and evaluate the efficacy of the treatment. In vivo testing showed that animals treated with our MIFU device improved on the retinal tortuosity and clinical dilation compared to the control group while evaluating fluorescein angiogram (FA) Images.

## 1. Introduction

Diseases affecting the posterior segment of the eye, such as age-related macular degeneration (AMD), diabetic retinopathy, retinal vein occlusion, and posterior uveitis, are difficult to treat because of the challenge of delivering therapeutically relevant concentrations of medication into the vitreous humor (VH) and retinal tissue [1]. Although topical eye drops are safe, this route of delivery is incapable of achieving therapeutic levels in the human VH required to exert their pharmacologic activity [2]. To ensure therapeutic concentrations, most drugs used to treat retinal conditions such as AvastinTM, LucentisTM and EyleaTM are directly injected into the posterior segment as an intravitreal (IVT) injection [3]. To maintain therapeutic concentrations, these agents must be given monthly or bimonthly. However, frequent IVT injections can predispose these patients to rare but severe complications like endophthalmitis, retinal detachment, increased intraocular pressure, cataractogenesis, and increased retinal ganglion cell apoptosis [4]. Thus, the ability to noninvasively deliver effective agents may reduce potential complications while improving treatment adherence.

Transscleral delivery is a novel strategy to efficiently achieve therapeutic level of drugs in the retina and choroid tissue by exploiting the large scleral surface area [5]. Despite the intrinsic permeability characteristics of the sclera, it continues to be a challenge to deliver high molecular weight (MW >70 KDa) compounds into the posterior segment [1]. Efforts to exploit this strategy include transscleral iontophoresis [6], which uses an electric field to deliver drugs into the retina and choroidal tissue. However, iontophoresis may be limited to charged drugs. Unfortunately, this procedure is not well tolerated by patients. Therefore, there is a need to develop a delivery system that can be used across a large spectrum of therapeutic agents and that is independent of its molecular weight.

In this paper, we developed a method for delivering antiinflammatory agents of high molecular weight (e.g., Avastin) into the posterior segment that is less invasive than IVT injections. Our novel delivery method employs moderate intensity focused ultrasound [MIFU]) to mediate scleral thinning and thus enhance drug transport across this barrier. To evaluate the efficacy of our delivery method, we developed a rabbit disease model using vascular endothelial growth factor (VEGF-A 165) to replicate the neovascular retinopathy.

## 2. Results

### 2.1. Transducer Performance Evaluation

#### 2.1.1. Basic Performance Evaluation

**Figure 1:**
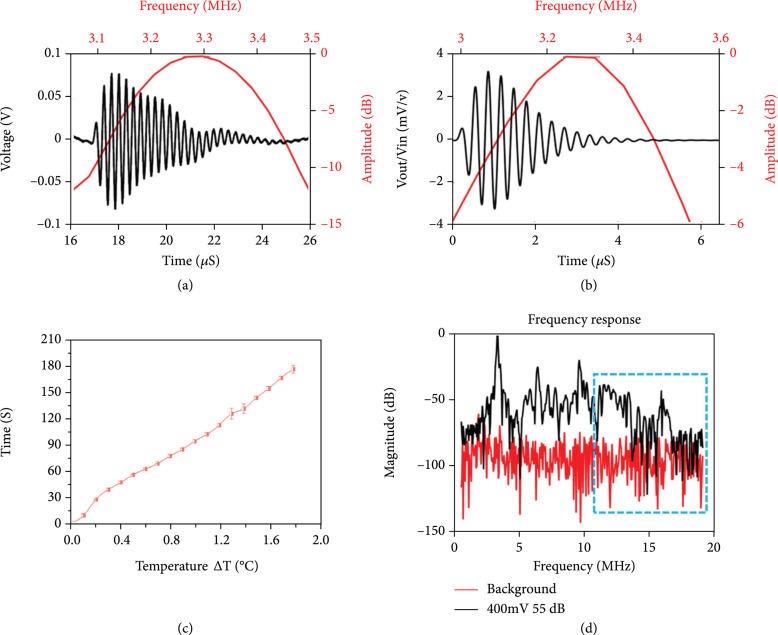
Summary of experimental transducer testing. (a, b) Comparison of experimental and simulated results in both time and frequency domain. (a) Measured pulse-echo response. (b) PiezoCAD simulation. (c) Temperature vs. time curve. The time points were recorded as the temperature increased in 0.1 °C intervals within a 3 min period. (d) Cavitation effect evaluation. The frequency response of the reflected ultrasound signal from the sclera under MIFU condition and background condition.

The center frequency of the transducer was measured as 3.27 MHz with the -6 dB bandwidth of 9%. The results indicated that it reached a good agreement with PiezoCAD simulation, where the center frequency was 3.25 MHz and the -6 dB bandwidth was 16% (Figures 1(a) and 1(b)).

**Figure 2:**
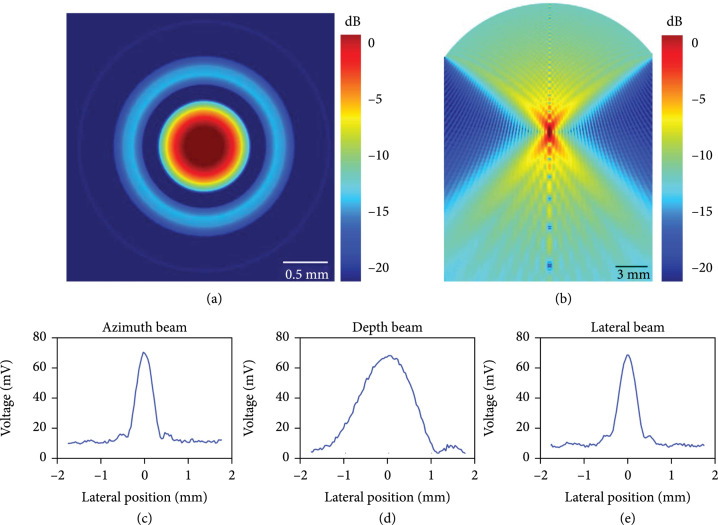
Hydrophone results and COMOSOL simulation results. (a) Simulated acoustic field in the *x*-*y* plane. (b) Simulated acoustic field in the *x*-*z* plane. (c-e) Measured beam in *x*, *z*, and *y* direction, respectively.

Hydrophone (HGL-0400, ONDA, CA, USA) measurement was conducted to characterize the acoustic field of the transducer (Figure 2). The acoustic pressure distribution was acquired as well as the lateral and axial beamwidth. The measured lateral and axial beamwidth were 0.663 mm and 1.872 mm, respectively. Finite-element analysis was conducted by COMSOL; the lateral and axial beamwidth was 0.778 mm and 1.554 mm, respectively.

#### 2.1.2. Thermal Effect Evaluation

The thermal effect caused by the interaction of the ultrasound and the sclera was measured with a needle-type thermocouple. The results showed that the temperature was increased 1.8 °C in total (Figure 1(c)).

#### 2.1.3. Cavitation Effect Evaluation

**Figure 3:**
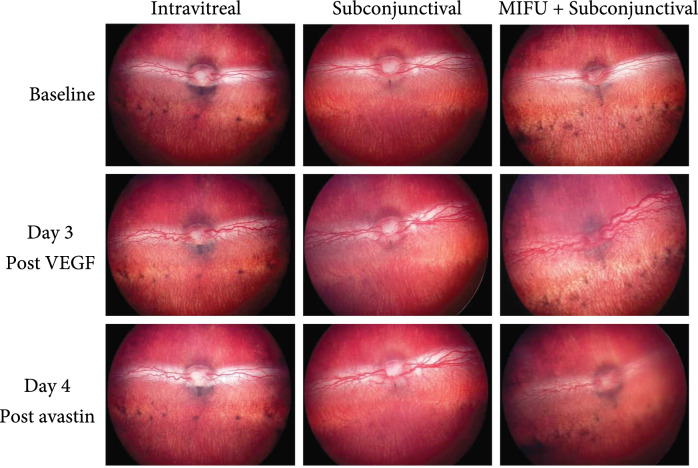
Fundus images of treated groups at day 0, day 3, and day 7. Top row shows baseline images where major blood vessels appear very straight with no tortuosity of small or large vessels (score: 0±0). Middle row shows fundus images at day 3 after VEGF injection and before Avastin treatment where frank tortuosity is present in major and minor vessels with retinal tractions. Bottom row shows fundus images at day 7 after VEGF injection and day 4 after Avastin treatment. Bottom left was treated with an intravitreal injection, bottom-middle was treated with a subconjunctival injection, and bottom right was treated with MIFU + subconjunctival injection.

The most commonly used passive cavitation detection (PCD) method was selected to evaluate the cavitation effect. The PCD system mainly contained 2 parts: detection transducer and emit transducer. The detection transducer had a center frequency of 15 MHz with a -6 dB bandwidth of 60%, and the sensitive frequency range of 11 MHz to 19 MHz was selected in this study. It was aligned confocally with the emit transducer guided by the hydrophone, and the sclera was placed in this focal spot. The detection transducer received the reflected ultrasound signal from the sclera. If the cavitation effect occurred, then a broadband shock wave would be generated due to the bubble collapsing. The cavitation effect was assumed to have occurred when the broadband noise caused by the shock wave in the frequency range mentioned above had more than √5 of amplitude comparing with the background signal [7, 8]. In Figure 3, despite the subharmonic signal at 16.35 MHz, the measured results met this criterion (Figure 1(d)).

### 2.2. Experimental Study

#### 2.2.1. MIFU Treatment

**Figure 4:**
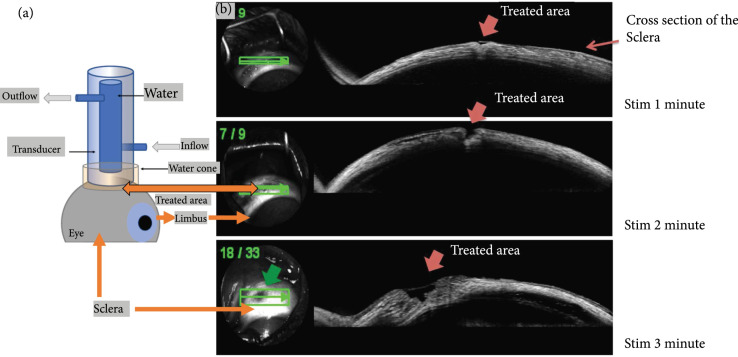
In vivo setup and sample OCT images of treated area. (a) This system allowed us to maintain MIFU intensity where the temperature is compatible with ocular temperature. (b) OCT images of the retina. Top image: sclera was treated with ultrasound for one min. Middle image: sclera was treated with ultrasound for two min. Bottom image: sclera was treated with ultrasound for three min.

MIFU treatment was applied at 3 different durations (1 min, 2 min, and 3 min) to evaluate and observe the changes caused in the sclera. Scleral thinning was observed for all three durations (Figure 4(b)). Since the objective of our study is to enhance large molecular therapeutic such as Avastin to transit through the barrier, it was determined qualitatively that 3 min MIFU treatment could maximally thin the sclera without disrupting structural integrity.

#### 2.2.2. Treatment Groups

**Figure 5:**
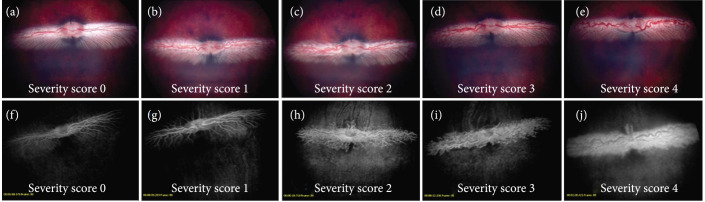
Fundus and FA images from Dutch belted rabbits. These images were used as a template for severity scoring based on observations. “Masked” evaluators quantified signs of tortuosity and neovascularization using a 0 to 4 severity scoring system.

**Figure 6:**
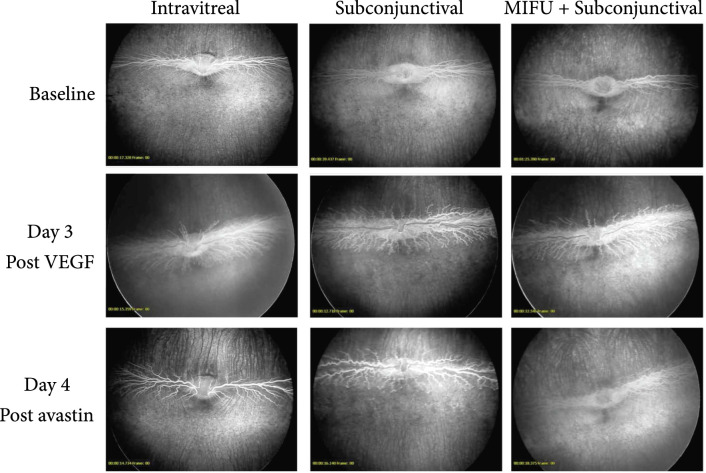
FA of treated groups at day 0, day 3, and day 7. Top row shows baseline images where major blood vessels appear very straight with no tortuosity of small or large vessels. Middle row shows FA images at day 3 after VEGF injection and before Avastin treatment where leakage tortuosity is present between major and minor vessels. Minor vessels are not visible. Bottom row shows FA images at day 7 after VEGF injection and day 4 after Avastin treatment. Bottom left was treated with an intravitreal injection, bottom-middle was treated with a subconjunctival injection, and bottom right was treated with MIFU + subconjunctival injection.

Retinal vascularization in all animals was scored using Figure 5. A severity scoring system from 0 to 4 was used to quantify signs of tortuosity and neovascularization, where 4 was defined as the most severe and 0 as normal. Vascularization was observed in all the rabbits at day 3 after VEGF induced CNV (score: 3.67±0.58). The injected eye showed signs of vasodilation and tortuosity and neovascularization (Figures 3 and 6). Rabbits in group 2 received an IVT injection of 1.25 mg Avastin 3 days after VEGF injection. 4 days after the avastin injection, animals showed a clinical and angiographic improvement (Score: 0 ± 0). On the contrary, animals from group 3 which received a subconjunctival injection of 1.25 mg Avastin 3 days after VEGF injection (Score: 3.33 ± 0.58) did not show retinal vascular changes 4 days post-avastin (3.33 ± 0.58). However, in NZB, receiving both MIFU treatment plus a subconjunctival injection of 1.25 mg Avastin showed an improvement on the retinal tortuosity and clinical dilation compared to their findings at day 3 (score at day 3: 3.67±0.58); FA images showed mild dilation with minimum leakage (score 4 days post Avastin: 1.33±0.58). Thus, group 4 shows a similar clinical effect compared to group 2. All animals from three different groups were scored using Figure 5 using a 0 to 4 severity scoring system, being 4 the most severe and 0 normal, and the results are summarized in Table 1.

**Table 1:** Scoring of all treated animals with Avastin. Retinal vascular changes were served among all rabbits at day 3 after VEGF injection. Rabbits with intravitreal injection of anti-VEGF drug (Avastin) showed a clinical and angiographic improvement at day 7. On the contrary, animals which received a subconjunctival injection did not show retinal vascular changes. MIFU group showed an improvement on the retinal tortuosity and clinical dilation compared to their findings at day 3. Scores are shown as mean±SD.

Scoring	Group 2: intravitreal injection	Group 3: subconjunctival injection alone	Group 4: MIFU + subconjunctival injection
Day 0	0±0	0±0	0±0
Day 3 post-VEGF injection	3.67±0.58	3.33±0.58	3.67±0.58
Day 7–day 4 post-Avastin	0±0	3.33±0.58	1.33±0.58

## 3. Conclusion and Discussion

In this study, a highly focused transducer was fabricated and utilized to mediate thinning of the sclera to enhance drug transport across this barrier. Being able to create a highly focused transducer allowed us to have a precise and focalized window of treatment. For safety reasons, it was important to validate that the thinning of the sclera was caused by the cavitation effect instead of the thermal effect. Temperature changes were evaluated during a three-minute trial of continued pulses, and an increase in temperature of 1.8 °C was observed during that time indicating a minimal thermal effect. Thus, it can be concluded that the thinning of the sclera was predominantly caused by the cavitation effect.

The cavitation bio effects can be quantified by means of a unitless measurement known as the mechanical index. In order to assess safety, the FDA has stipulated that the mechanical index of ultrasound devices used in the ophthalmology field for diagnostic purposes cannot exceed a value of 0.23. Although our current device has a mechanical index of 10, our study showed no adverse effects on other tissue structures besides the thinning of the sclera observed in the OCT images. Since our ultrasound device is meant to be used for treatment purposes, the FDA proposed safety limit does not directly translate to our application. Future studies should focus on finding mechanical index limits for the safe utilization of ultrasound with treatment purposes.

The high intensity focused ultrasound (HIFU) has been used by other groups in the ophthalmology field with treatment purposes in both clinical and preclinical applications [9, 10], and safety issues have rarely been reported [11]. For example, Razavi et al. used an ultrasound device with mechanical index ranging from 6.5 to 12.2 to modify the permeability of the sclera. This work showed that inertial cavitation enhances the permeability of the sclera with no significant alterations of the tissue observed in the electron microscopy images [12]. Aptel et al. utilized ultrasound coagulation of the ciliary body with HIFU to reduce intraocular pressure in both an animal study and a pilot human study. Their results showed that HIFU was well tolerated and no significant damage was observed in the histology images in the animal study [13]. Similarly, no major intraoperative or postoperative complications were reported in the human study [14]. The aforementioned results provide evidence that focused ultrasound can be used safely for treatment purposes in ophthalmology applications.

To test the efficacy of this device in vivo, we were able to successfully create a disease model using pigmented rabbits to mimic the behavior of retinal retinopathies by using VEGF-165. Other groups have also developed models with persistent retinal neovascularization by using either Dutch belted rabbits [15] or New Zealand rabbits [16] . This paper on the other hand created a retinal disease model that could be used for both breeds allowing us to compare the behavior of the VEGF-165A based on the different breed characteristics. This information will be useful for future research that aims at testing new protocols to treat retinopathies.

By using a disease model instead of normal pigmented rabbits, we were able to test the efficacy of the novel drug delivery system by objectively quantifying the Anti-VEGF medication (Avastin) effects on the retina. After comparing the control (subconjunctival injection only) versus the MIFU treated group (MIFU + subconjunctival injection) based on the FA images, we observed a definitive improvement in the tortuosity and neovascularization of the retina in the MIFU group, while no improvement was observed in the control group. This allowed us to conclude that sufficient concentration of medication penetrated the sclera when the MIFU device was used.

We have shown that the scleral thinning obtained by the MIFU treatment can facilitate the penetration of large molecules across the scleral barrier. Our pilot study has demonstrated that there is a negative correlation between the permeability of the sclera and overall thickness. Despite the fact that the rabbits’ sclera is thinner compared to humans’ sclera, we believe that the observed correlation may still apply to humans. This work has shown promising evidence that this is a viable method to deliver high molecular weight medications not invasively. It should be noted that our in vivo results are based on a small sample size and only assessed the thinning of the sclera immediately after treatment. Our future work will focus on further testing safety and efficacy of our device in a larger sample size and studying the long term permeability of the treated area for transscleral transport, since currently, patients receive multiple Avastin treatments for long periods of time.

## 4. Materials and Methods

### 4.1. Transducer

#### 4.1.1. Experimental Setup

For the in vivo studies, a PZT-4 based transducer with an aperture size of 20 mm and the focal length of 13 mm was fabricated. Parylene C (Specialty Coating Systems, Indianapolis, IN, USA) was used to compensate for the acoustic mismatch between the piezoelectrical material and the water, and it also served as a water insulation layer [17]. Air was used as the backing material to enable max acoustic power output [18].

A water-cooling system circulates water through the transducer housing, removing the heat when the transducer is working, allowing us to use it for longer periods of time during in vivo study. The focused ultrasound passes through the small hole at the bottom of the water cone allowing us to focus accurately on the sclera (focal spot) (Figure 4(a)).

The transducer was held by a five-axis translational stage to enable a perpendicular ultrasonic beam to the sclera. The transducer was driven by a function generator (AFG 3252 C, Tektronix, Beaverton, OR, USA) and a RF amplifier (100A250A, Amplifier Research, PA, USA) with 55 dB of gain. The ultrasonic transducer was placed on the focal spot. In order to select the optimal stimulation time, various pulse durations (1, 2, and 3 min) were evaluated in vivo using OCT images (Figure 4(b)). The size and depth of the scleral thinned area after treatment were used to select the ideal stimulation duration. The mechanical index was set around 10 with duty cycle of 12%. Table 2 summarizes the waveform generator parameters used during MIFU stimulation.

**Table 2:** Waveform generator parameters. The parameters described above were used during all in vivo experiments.

Waveform generator parameters	
Waveform	Sinewave
Peak to peak voltage	0.4Vpp
Duty cycle	400 cycle: burst
Pulse period	1 ms burst period
Output frequency	3.3 MHz

#### 4.1.2. Performance Evaluation Tests

To evaluate the performance of the transducer, several characterizations have been made as followed: pulse-echo test and PiezoCAD simulation, hydrophone test and COMOSOL (COMSOL 5.3a, COMSOL Inc., MA, USA) simulation, thermal effect, and cavitation effect mechanism investigations. Pulse-echo measurement was described in our previous work with the same procedures [19], and hydrophone measurement was also provided in our previous work [20]. The investigation of the thermal effect was achieved by a thermal couple. The sclera was positioned on the focal spot of the transducer, and a lead was inserted under the sclera shell. Every 0.1 °C of temperature raised, the corresponding time point was recorded, and the total time span was 3 min. The cavitation effect was conducted by a confocally transmit transducer and a receive transducer. The received transducer recorded the reflected echo from the sclera; the echo signal was saved for offline processing.

### 4.2. Animal Model

Rabbits share anatomical and physiological similarities with the human eye. Ocular size, volume, internal features, and their diffusional path to reach the anterior segment using topical administered compounds are some of them, making it a great model for our purpose [21].

All animals were maintained on a daily 12 hr light/dark cycle. All procedures were in conformance with the Guide for Care and Use of Laboratory Animals (National Institutes of Health). The University of Southern California Institutional Animal Care and Use Committee (IACUC) reviewed and approved all procedures before commencement of the study.

We performed survival experiments during our study. Adult New Zealand Black (NZB) rabbits (Western Oregon), ~2-3 kg, were used (n=12). All experiments were performed in the right eye of each animal. The animals were anesthetized with ketamine (100 mg/kg) and xylazine (20 mg/kg) combination and euthanized at the end of the study.

#### 4.2.1. Disease Model

Retinopathies are characterized by the development of choroidal neovascularization (CNV) through endogenous activation of VEGF elaboration [22, 23]. To validate the efficacy of the drug delivery system, a disease model replicating the behavior of retinal retinopathies was created. The creation of this model allowed us to test anti-VEGF medications and evaluate the efficacy of the treatment.

To find the correct concentration of VEGF-A 165 that is needed to create our disease model, rabbits from two different breeds (New Zealand black and pigmented Dutch belted) were stratified into two different groups to receive either 1 *μ*g or 2 *μ*g of freshly reconstituted VEGF-A 165 intravitreally. Ophthalmologic examinations were conducted on days 7 and 14 to determine the severity as well as the resolution of the retinal edema and angiogenesis at designated time points. At the end of the study (EOS) or day 14, the fundus and fluorescein angiography (FA) images were scored by a “Masked” evaluator for signs of tortuosity and neovascularization using a 0 to 4 severity scoring system (Figure 5).

### 4.3. Experimental Study

The eye was dilated with two drops of 1% tropicamide (Tropicacyl, Akorn Inc., Buffalo Grove, IL) and 2.5% phenylephrine (AK-Dilate, Akorn Inc., Buffalo Grove, IL). The animal’s heart rate, blood pressure, temperature (rectal), and respiratory rate were monitored and recorded every 5 minutes during surgical procedure and recovery time for survival experiments. The animal’s body temperature was maintained at 37 °𝐶 with an electric heating pad.

The VEGF-A 165 (R&D systems; catalogue number 293-VE-050/CF) was freshly reconstituted at a concentration of 3 *μ*g/50 *μ*L. On day 1, baseline ocular examination, fundoscopic images, and fluorescein (FA) image were taken. The rabbits received 50 *μ*l as an intravitreal injection of VEGF-A into the right eye.

On day 3, ophthalmologic examinations using funduscope and fluorescein angiography (FA) were used to assess the severity of the angiogenesis and retinal edema (Figure 5). The transducer was held by a three-axis translational stage (Model 4044 M Parker Daedal, Cleveland, OH) mounted on a magnetic based articulating arm for precise placement. The transducer was placed on the surface of the sclera ~3 mm from the limbus without applying any pressure to it. On day 7, at the conclusion of the study, all animals were euthanized with an intravenous injection of pentobarbital (30 mg/kg; Butler, Dublin, OH).

#### 4.3.1. Treatment Groups

Four treatments groups were studied (Table 3). The fundus images and fluorescein images were acquired for all groups at day 0, day 3, and day 7.

**Table 3:** Experimental groups of study. SC = subconjunctival, EOS = end of study, D0 = day 0, D3 = day 3, D7 = day 7.

ID	Treatment	N	VEGF	Avastin	MIFU	EOS
Control	VEGF (3 *μ*g)	3	D0	NA	NA	D7
Control 1	VEGF (3 *μ*g) + Avastin (1.25 mg) IVT	3	D0	D3	NA	D7
Control 2	VEGF (3 *μ*g) + Avastin (1.25 mg) SC	3	D0	D3	NA	D7
Treatment	VEGF(3 *μ*g) + MIFU(3 min) + Avastin(1.25 mg) SC	3	D0	D3	D3	D7

*(1) Group 1: VEGF Control*. 3 rabbits received an intravitreal injection of 3 *μ*g/50 *μ*L of VEGF into the right eye and followed up at day 3 and day 7 to study the VEGF behavior without treatment.

*(2) Group 2: VEGF + Intravitreal (IVT) Avastin Only*. At day 0, 3 rabbits received an intravitreal injection of 3 *μ*g/50 *μ*L of VEGF into the right eye. At day 3, all animals received an intravitreal injection of Avastin (1.25 mg/50 *μ*L) in the treated eye. At day 7, ophthalmologic examinations using funduscope and fluorescein angiography (FA) were used to assess control treatment group.

*(3) Group 3: VEGF + Subconjunctival Avastin Only*. At day 0, 3 rabbits received an intravitreal injection of 3 *μ*g/50 *μ*L of VEGF into the right eye. At day 3, all animals received a subconjunctival injection of Avastin (1.25 mg/50 *μ*L) in the treated eye. At day 7, ophthalmologic examinations using funduscope and fluorescein angiography (FA) were used to assess control treatment.

*(4) Group 4: VEGF + MIFU + Subconjunctival Avastin*. At day 0, 3 rabbits received an intravitreal injection of 3 *μ*g/50 *μ*L of VEGF into right eye. At day 3, all animals received MIFU treatment for 3 minutes (settings summarized in Table 1) and a subconjunctival injection of Avastin (1.25 mg/50 *μ*L) in the right eye on top of treated area. At day 7, ophthalmologic examinations using funduscope and fluorescein angiography (FA) were used to assess treatment.

## Data Availability

Data is available upon request from the authors.
